# Learned Motor Patterns Are Replayed in Human Motor Cortex during Sleep

**DOI:** 10.1523/JNEUROSCI.2074-21.2022

**Published:** 2022-06-22

**Authors:** Daniel B. Rubin, Tommy Hosman, Jessica N. Kelemen, Anastasia Kapitonava, Francis R. Willett, Brian F. Coughlin, Eric Halgren, Eyal Y. Kimchi, Ziv M. Williams, John D. Simeral, Leigh R. Hochberg, Sydney S. Cash

**Affiliations:** ^1^Center for Neurotechnology and Neurorecovery, Department of Neurology, Massachusetts General Hospital, Boston, Massachusetts 02114; ^2^Harvard Medical School, Boston, Massachusetts 02114; ^3^Center for Neurorestoration and Neurotechnology, Department of Veterans Affairs, Providence, Rhode Island 02908; ^4^Carney Institute for Brain Science and School of Engineering, Brown University, Providence, Rhode Island 02912; ^5^Hughes Medical Institute at Stanford University, Palo Alto, California 94305; ^6^Departments of Neurosciences and Radiology, University of California at San Diego, La Jolla, California 92093; ^7^Department of Neurosurgery, Massachusetts General Hospital, Boston, Massachusetts 02114; ^8^Program in Neuroscience, Harvard-MIT Program in Health Sciences and Technology, Harvard Medical School, Boston, Massachusetts 02115

**Keywords:** brain computer interface, learning, memory, replay, sleep

## Abstract

Consolidation of memory is believed to involve offline replay of neural activity. While amply demonstrated in rodents, evidence for replay in humans, particularly regarding motor memory, is less compelling. To determine whether replay occurs after motor learning, we sought to record from motor cortex during a novel motor task and subsequent overnight sleep. A 36-year-old man with tetraplegia secondary to cervical spinal cord injury enrolled in the ongoing BrainGate brain–computer interface pilot clinical trial had two 96-channel intracortical microelectrode arrays placed chronically into left precentral gyrus. Single- and multi-unit activity was recorded while he played a color/sound sequence matching memory game. Intended movements were decoded from motor cortical neuronal activity by a real-time steady-state Kalman filter that allowed the participant to control a neurally driven cursor on the screen. Intracortical neural activity from precentral gyrus and 2-lead scalp EEG were recorded overnight as he slept. When decoded using the same steady-state Kalman filter parameters, intracortical neural signals recorded overnight replayed the target sequence from the memory game at intervals throughout at a frequency significantly greater than expected by chance. Replay events occurred at speeds ranging from 1 to 4 times as fast as initial task execution and were most frequently observed during slow-wave sleep. These results demonstrate that recent visuomotor skill acquisition in humans may be accompanied by replay of the corresponding motor cortex neural activity during sleep.

**SIGNIFICANCE STATEMENT** Within cortex, the acquisition of information is often followed by the offline recapitulation of specific sequences of neural firing. Replay of recent activity is enriched during sleep and may support the consolidation of learning and memory. Using an intracortical brain–computer interface, we recorded and decoded activity from motor cortex as a human research participant performed a novel motor task. By decoding neural activity throughout subsequent sleep, we find that neural sequences underlying the recently practiced motor task are repeated throughout the night, providing direct evidence of replay in human motor cortex during sleep. This approach, using an optimized brain–computer interface decoder to characterize neural activity during sleep, provides a framework for future studies exploring replay, learning, and memory.

## Introduction

The neurobiological function of sleep remains incompletely characterized; however, several compelling lines of evidence suggest it plays an essential role in learning and memory. Offline replay of recently learned neural sequences during sleep supports the teleological theory of sleep for neural rehearsal/practice, and comports with the Hebbian model of learning and plasticity (Huber et al., 2004; Abel et al., 2013; Tononi and Cirelli, 2014). Replay of learned neural sequences during sleep has been demonstrated in rat hippocampus (Wilson and McNaughton, 1994; Skaggs and McNaughton, 1996; Foster and Wilson, 2006; Davidson et al., 2009) and sensory cortex (Ji and Wilson, 2007), and optogenetic manipulation of the electrophysiologic components of sleep architecture that support replay influences subsequent task performance (Gulati et al., 2014; van de Ven et al., 2016; Kim et al., 2019).

Slow-wave sleep (SWS, in humans referred to as non-rapid eye movement Stages 3 and 4 [NREM3/4]) is particularly important in these processes, as SWS is enriched in the electrophysiologic phenomena that support neural replay. In hippocampus, replay of neural sequences occurs during bursts of activity called sharp-wave ripples (SWRs) (Wilson and McNaughton, 1994; Carr et al., 2011, 2012; Singer et al., 2013; Papale et al., 2016). SWRs are predominant during SWS (Miyawaki and Diba, 2016) but can also be observed in the awake state, where they are closely associated with task performance (Karlsson and Frank, 2009; Carr et al., 2011; Singer et al., 2013; Vaz et al., 2020).

Human studies similarly support a replay model of memory consolidation during SWS (Huber et al., 2004). Hippocampal replay during SWS of recently learned neural sequences has been demonstrated with FDG-PET and fMRI and by intracortical recording with electrocorticographic and depth recordings (Peigneux et al., 2004; Deuker et al., 2013; Jiang et al., 2017; Schapiro et al., 2018; Zhang et al., 2018). Cortical motifs of neural activation identified during wakeful cognitive task performance are enriched in hippocampal SWR, neocortical spindles, and neocortical down-to-up transitions in subsequent sleep in a coordinated fashion that supports memory consolidation during SWS (Jiang et al., 2017, 2019).

Relatively little is known about the role of replay in motor cortex. Previously, our group demonstrated indirect evidence of replay of recently learned neural signals underlying a novel motor task in human motor cortex (Eichenlaub et al., 2020). However, this study was technically limited by an inability to record during overnight sleep; thus, no SWS data could be obtained. A recent study in nonhuman primate motor cortex did show partial preservation of the order of activation of neurons that fire during transition to “up” states in SWS compared with the order of activation during waking behavior (Xu et al., 2019). As this work did not characterize the awake neural activity during specific behaviors, the connection between these findings and task-specific motor activation remains speculative. In the present study, we used a wireless intracortical microelectrode brain–computer interface (BCI) recording preparation paired with wireless surface EEG technology to assess for neural replay of a novel motor task during SWS in a human research participant (Simeral et al., 2021). By using an optimized BCI decoding algorithm as a dimensionality reduction tool, we demonstrate, for the first time, direct evidence of replay in human motor cortex during sleep. In addition to revealing a novel feature of human sleep, this methodological approach may be useful for future studies assessing the impact of various manipulations on neural and behavioral measures of learning and memory.

## Materials and Methods

### Data/code availability

Any data reported in this paper will be shared by the corresponding author on reasonable request. All original code has been deposited at Github and is publicly available as of the date of publication at the following url: https://github.com/drubin4/Replay_in_Sleep_Motor_Cortex. Any additional information required to reanalyze the data reported in this paper is available from the corresponding author on request.

### Participant details, data acquisition

Permission for this study was granted by the U.S. Food and Drug Administration (Investigational Device Exemption #G090003) and the Institutional Review Boards of Massachusetts General Hospital, Providence VA Medical Center, and Brown University. The research participant (identified as T11) gave informed consent to the study and publications resulting from the research. Neural recording took place during two separate multiday sessions. Details of the recording paradigm have been described previously (Hochberg et al., 2006, 2012; Malik et al., 2011; Eichenlaub et al., 2020). Briefly, a 36-year-old right-handed man with a history of tetraplegia secondary to a cervical spinal cord injury that occurred 11 years prior had two 96-channel intracortical microelectrode arrays placed chronically into the left precentral gyrus (PCG) as part of the ongoing BrainGate pilot clinical trial (www.ClinicalTrials.gov; Identifier: NCT00912041). Array placement was performed 153 d before the first recording session and 427 d before the second recording session. Throughout the period before, between, and after the research sessions described herein, the participant has taken part in approximately twice-weekly research sessions for other neuroscientific and engineering studies and had frequent practice using the BrainGate neural interface system to control a computer cursor.

During each recording session, single-unit and multiunit activity was recorded at 30 kHz from each of the 192 channels and processed through a custom neural signal processing system. Activity was digitally downsampled and processed in real time to yield two metrics of neuronal activity from each channel, power within the spike band (250-5000 Hz) frequency range as well as threshold crossing events, which are collectively referred to here as neuronal activity. Metrics are calculated within 20 ms time-steps. Using a standardized calibration routine, these features were used to encode the parameters of a steady-state Kalman filter used for closed loop cursor control (Malik et al., 2011). With this encoding schema, the participant had facile control over a neurally driven cursor on the screen (Brandman et al., 2018), which was subsequently used in the experimental task sessions. In separate offline analysis, automated spike sorting based on waveform shape and amplitude was performed to evaluate the nature of the signal components contributing to the neuronal activity recorded (Vargas-Irwin and Donoghue, 2007).

In addition to intracortical recording, during each recording session, EEG was recorded using a battery powered, single-channel device (Epilog, Epitel). To facilitate scalp recordings, the device was connected to standard gold cup electrodes (Natus Grass Surface Electrode discs, Natus Medical), which were affixed with the device to the participant's scalp to allow for continuous EEG recording. Leads were placed at approximately C3 and Cz (as defined by the international 10–20 system for EEG recording), within the spatial constraints inherent in the device lead length and intracortical electrode pedestal position. EEG signals were acquired at 512 Hz and preprocessed with a third-order Butterworth bandpass filter between 0.5 and 50 Hz before analysis.

### Experimental paradigm

At the beginning of each recording session, a short synchronization routine was performed to align the internal clocks of the wireless surface EEG device and the intracortical neural signal processors. Briefly, on initiation of the synchronization routine, the neural signal processor was triggered to produce an audible tone while adding a high-amplitude square wave impulse to the neural recording. This audible tone served as a cue for the researcher, who tapped 3 times on one of the surface EEG leads. This synchronization routine was repeated before each daytime task performance session, before each overnight recording session, and at the end of each overnight recording session. In later offline analysis, the square-wave impulse present on the intracortical recording was aligned with the positive deflections produced by the tapping artifact on the surface EEG recording, thus aligning the internal clocks of the two systems (within the margin of error of the reaction time of the researcher). Following alignment of the internal clocks prior to the time of the first synchronization routine, the internal clocks remained aligned at subsequent synchronization times within the same multiday recording event, indicating that there was not a significant drift between the internal clocks of the two recording systems.

Following the first synchronization routine, the participant then completed a center-out calibration task to optimize the parameters of the steady-state Kalman filter that would be used for all the subsequent experimental tasks for the day (Malik et al., 2011; Brandman et al., 2018). This procedure has been described previously but is summarized as follows. Calibration is performed to identify the parameters of the steady-state Kalman filter that maximize the joint probability of a set of movement directions and associated set of firing rates. To do so, the participant is asked to complete a radial-eight center-out task. On each trial, the participant is cued to one of eight targets surrounding a center fixation point and is asked to attempt to control the cursor to move it from the center out to the target, and to dwell the cursor on the cued target for 1 s. Intended movement directions (inferred by target location under the assumption that the participant is faithfully attempting the task) are regressed against the corresponding neural activity (inclusive of both spike band power and threshold crossing events on each channel) to obtain the best-fit parameters of the steady-state Kalman filter using the 40 feature channels (again inclusive of both spike band power and threshold crossing events) that have the greatest modulation with movement direction. An error attenuation (EA) function is used to facilitate control during the initial phase of calibration. An EA of 1.0 indicates that all error is “removed” from the neural signal and the cursor moves automatically from the center to the target, and an EA of 0 indicates that the participant has complete control over the cursor position. In this calibration routine, EA is decreased from 0.9 to 0.0 in a stepwise fashion over the first 90 s of the 5 min session, and the participant has complete control over the cursor for the remaining 3.5 minutes. Kalman filter weights are updated iteratively over the course of the calibration session (Brandman et al., 2018).

**Figure 1.**
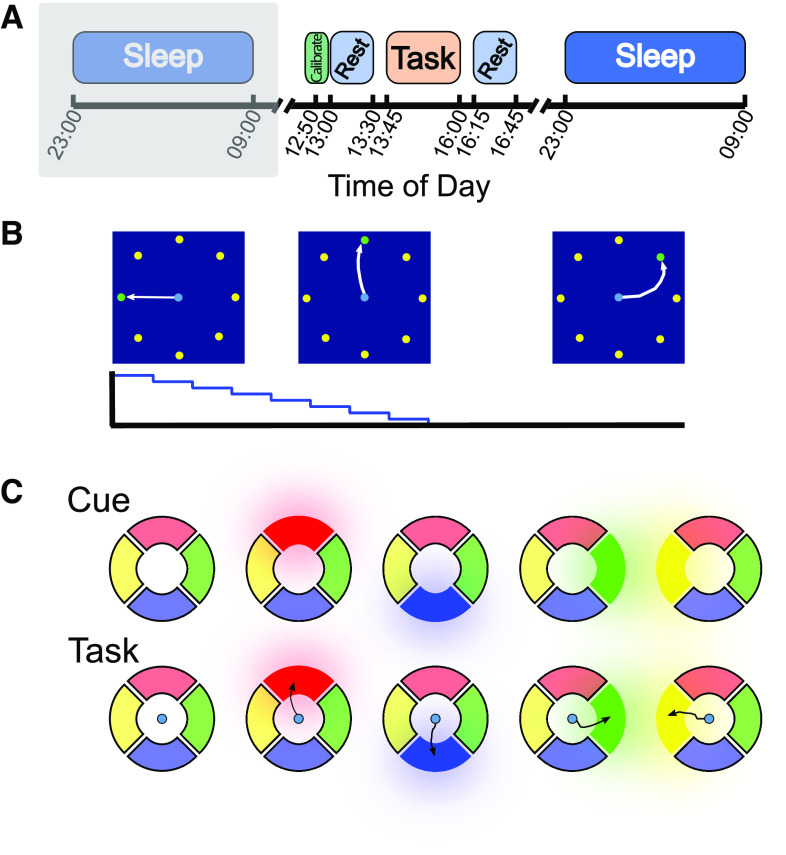
Overview of experimental paradigm. ***A***, Timeline of the recording sessions. On the second of the two recording dates, we recorded intracortical neural data and surface EEG during the night before the experimental session. Both recording sessions then otherwise began with a brief intracortical/EEG sync routine, followed by the standard calibration routine. ***B***, During the calibration routine, the participant completes a radial-eight center-out task, during which he is cued to move the cursor to 1 of 8 targets surrounding the center starting point. Early in the calibration routine, the error attenuation (EA) (on the accompanying graph) is set close to 1, such that most of the cursor movement is performed automatically. The internal weights of the steady-state Kalman filter are iteratively updated as the EA is reduced in a stepwise fashion and the participant is given full control of the neurally driven cursor. After calibration, the participant then took a 30 min rest during which we recorded both intracortical and surface signals. Following the rest period, the participant engaged in the motor sequence memory task (detailed in ***C***). After 10 blocks of the motor task, the participant took another 30 min rest. We then recorded neural signals overnight as the participant slept. ***C***, The motor sequence memory task. Every trial begins with a sequence demonstration, during which 1 of the 4 colored shapes is sequentially illuminated as a brief auditory tone is played. This sequence is played over 3 s. After the sequence demonstration is complete, a small neurally controlled cursor appears in the middle of the screen. The participant's task is to move the cursor to each of the 4 shapes in the same sequence as was just demonstrated. The participant completed 10 blocks of 16 trials on each recording session day. On each recording session day, there was one “target” sequence (which was different between the two sessions). In each block, 12 of the 16 trials were of the target sequence, and the other 4 trials were randomly generated “distractor” sequences.

After a 30 min rest period during which baseline neural activity was recorded, the participant was given the instructions for the memory matching task (see Fig. 1). This task is modeled on the handheld electronic game “Simon” https://en.wikipedia.org/wiki/Simon_(game). On each trial, the participant was cued by the sequential illumination of one of the four brightly colored targets on the screen. Each illumination lasts 400 ms and is accompanied by a distinctive tone. The participant was instructed to imagine moving his right hand to drive the neurally controlled cursor from the center of the screen out to each target in the same order as was just presented. If the participant dwells the cursor on the correct target for 300 ms, the acquisition is successful, and the cursor is recentered at the origin. When the participant successfully completes the sequence of four target acquisitions, a bright tone indicates success and the next trial's sequence is presented. If the cursor is dwelled on an incorrect target or if the participant fails to move the cursor to any target within 5 s, a low-pitched tone indicates trial failure and the next trial is started. Each sequence always uses all four polygons exactly once. Each block consists of 16 trials, with one sequence per trial. In any given block, 12 of the 16 trials are a set “target” sequence, and the other four trials are of a randomly chosen distractor sequence. The target sequence was different on the two multiday recording sessions but within a given session was consistent across all blocks; the distractor sequences were any of the other 23 possible four-target sequences. The participant was not told that there is a target sequence; distractor sequences were included both to maintain the participant's engagement in the task as well as to allow for tuning of the template matching thresholds (described in Quantification and statistical analysis*)*. The participant completed 10 blocks on each day, for a total 160 trials (of which 120 were of the target sequence). Between each block, the participant was given the opportunity to pause or take a short break if needed before proceeding to the next block. Following the 10 blocks of the motor sequence memory task, the participant was again asked to close his eyes for 30 min and invited to nap.

The evening after performing the blocks of the motor memory task, we recorded neural activity overnight as the participant slept. After the participant got into bed, the wireless intracortical transmitters and surface EEG were reattached, and the two devices were once again synced. Intracortical and surface EEG data were recorded throughout the night as the participant slept. For the second of the two multiday recordings, neural activity during overnight sleep was also recorded the night before the day of the motor memory task.

### Quantification and statistical analysis

We extracted spiking data (spike band [250-5000 Hz] power and threshold crossing events) for the duration of the two multiday recording sessions from each of the 192 intracortical channels. We then processed spiking data through the steady-state Kalman filter using the parameters fit during that session's calibration routine to generate a time series of “predicted” cursor coordinates, predicted in that during the resting and sleeping epochs, which comprise the majority of the recording sessions, no cursor is displayed on a screen.

From the data collected during 10 blocks of game play, we averaged the trajectories of hypothetical cursor coordinates produced by the steady-state Kalman filter during each successful target sequence trial to create a pair (one for each spatial dimension of cursor movement) of idealized task completion “template trajectories.” We used these templates to probe for evidence of replay during the resting and sleeping epochs as follows. At each time-step of the neural recording, for each of the two spatial dimensions, we calculated the cross-correlation between the template and the output of the steady-state Kalman filter. This process yielded two time-series of correlation coefficients equal in length to the time series of the neural recording. Separately for the X- and Y-dimensions, the 98th (for Session 1) or 99th (for Session 2) percentile of the correlation coefficients was chosen as the threshold to designate an activity pattern as a template match. We designated instances when the correlation crossed threshold in both the X- and Y-dimensions simultaneously as simultaneous threshold crossing events (STCEs). STCEs occurring over neighboring time-steps are classified as a single event. STCEs occurred tautologically during the awake task performance blocks. When occurring during rest or sleep, we refer to these instances as putative replay events. The specific percentile implemented as the threshold for a session was selected to optimize the performance of STCEs to correctly identify successful target trials and not identify unsuccessful target and all distractor trials during the active task performance. This was quantitatively operationalized by finding the integer percentile that jointly maximized the sensitivity and specificity (i.e., the Youden's J statistic: sensitivity + specificity − 1) of STCEs to accurately identify successful target trials during awake task performance.

To evaluate whether there is relative preservation of neuronal firing sequence during these putative replay events, we determined the order of neuronal firing during each successful target trial and each putative replay event by calculating, for each channel (representing the single or multiunit activity recorded), the time bin within 4 s after the onset of the task completion or replay event that had the maximum firing rate. Thus, for each event (task performance or putative replay), a 96-element sequence was identified. To determine the preservation of firing order across events, we calculated the pairwise matching index I_m_ between each task completion and putative replay event, using the approach derived by Ji and Wilson (2007) where I_m_ is defined as follows. For an M-channel recording, there are M(M – 1)/2 pairs of channels; between two events, let *m* be the number of pairs that have the same order of peak firing between the two events, and *n* be the number of pairs that have the opposite order. Define I_m_ = (m – n)/(m + n), such that I_m_ is bounded by [−1, 1]. Two events with precisely the same sequence of activation will have I_m_ = 1 and two events with exact opposite order of activation would have I_m_ = −1. To determine whether the distribution of matching indices we observe are greater than would be expected by chance, we generated a control distribution by calculating the matching indices of 100,000 pairs of randomly generated 96-element-long sequences. We limited this analysis to activity on the medial array, as we found activity on the lateral array to be sparser and less frequently associated with task completion.

To determine whether the number of putative replay events observed overnight exceeded the number that would be expected by chance, we used the following pair of bootstrap controls. As a first control, we wished to generate a distribution of the number of putative replay events expected for any randomly selected, nonbehaviorally salient, epoch of neural data. To do so, we randomly selected 100 segments of neural activity, each equal in duration to the successful target sequence templates, from the 30 min period of rest recorded immediately before the task blocks. We used the output of the Kalman filter generated by these randomly selected segments to produce a series of 100 pseudo-templates. For each pseudo-template, we repeated the template-matching procedure described above, calculating the cross-correlation between the template and the Kalman filter output at each time-step of the recording, and counted the number of STCEs for each pair of pseudo-templates. Because the generated distribution was highly left-skewed, we used a Wilcoxon rank-sum test to compare this distribution with our observed outcome from the true target template to determine whether the number of observed putative replay events was greater than would be expected by chance (i.e., compared with the distribution of STCEs produced from the pseudo-templates).

Because the random “pseudo-template” control described above did not necessarily preserve neural firing rate statistics, as a second control, we performed an alternative bootstrap procedure that specifically preserved the statistics of the underlying neural firing. In this control, for each of 100 iterations, the Kalman filter output for the duration of the recording was broken into 5 min segments. Within each segment, we used the discrete Fourier transform to randomize the phase of the X and Y dimension of the Kalman filter output. We then reassembled the segments into full-time-series and performed the same cross-correlation matching procedure described above using the true target templates. In this way, the bootstrapping process yields a distribution of STCEs that would be expected if only the unstructured statistical subspace of firing rates, but not the actual sequence of neural activation, was replayed overnight.

For the purpose of demonstration, several of the putative replay events were extracted, and the 2D trajectories predicted by the Kalman filter model were reconstructed into movies to demonstrate the cursor trajectories that would have been observed had the screen cursor and Simon game been active while the participant slept.

To assess for neuronal replay of target trajectories at different speeds, for each recording session, we used cubic splines to fit the pair of target Kalman filter trajectories and then adjusted the duration of the template using a temporal dilation/compression factor we define as τ. We varied τ over 18 values from 0.1 to 10. For each value of τ, the number of STCEs was calculated during each epoch as above. We ran the phase-randomized bootstrapping control at each value of τ to assess for statistical significance.

To quantitatively stage sleep and examine the relationship between putative replay events and sleep stage, we derived a simple metric of dominant EEG frequency by calculating the 95% frequency power limit (FPL) of the EEG signal. To do so, at each time-step of the EEG frequency spectrogram, the power of the normalized spectrogram was cumulatively summed, and the frequency at which the cumulative sum first exceeded 0.95 was the FPL. During the overnight portions of the recording, each interval of sleep (i.e., each time-step of the EEG spectrogram) was categorized as either SWS or non-SWS, depending on whether it fell below or above a FPL threshold. For quantitative analyses relating sleep stage to intracortical data, the FPL threshold used to define SWS was varied systemically from 2.0 to 6.0 Hz to ensure the robustness of all findings.

We characterized ripple activity during sleep through a pair of quantitative approaches. As a first-pass approximation of ripple activity, we calculated the power of the local field potential (LFP) signal within the low γ range (∼40-125 Hz); average power within this “ripple-band” was *z*-scored (within a 1 min sliding window) and used to identify epochs within which putative ripple activity would be expected. To then more precisely identify cortical ripples, we adopted the technique of Jiang et al (Jiang et al., 2019; Dickey et al., 2021b). To summarize briefly, signals from each channel were downsampled to 1000 Hz and bandpass filtered at 60-100 Hz using a zero-phase IIR filter. The root mean square (RMS) of the filtered signal using a 20 ms moving average was calculated, and segments in which this RMS value exceeded the 90th percentile of values were taken as putative ripples. Neighboring putative ripple segments that were <40 ms were merged into a single putative ripple. Each putative ripple that was at least 40 ms in duration was then evaluated for the number of distinct peaks in the LFP signal (after lowpass filtering at 120 Hz); a putative ripple was confirmed if it contained at least three distinct peaks within a single 40 ms window and the first and last peak of these peaks were not both within 7 ms from the edge of 40 ms time bin.

## Results

### Experimental task was rapidly acquired and successfully performed

**Figure 2.**
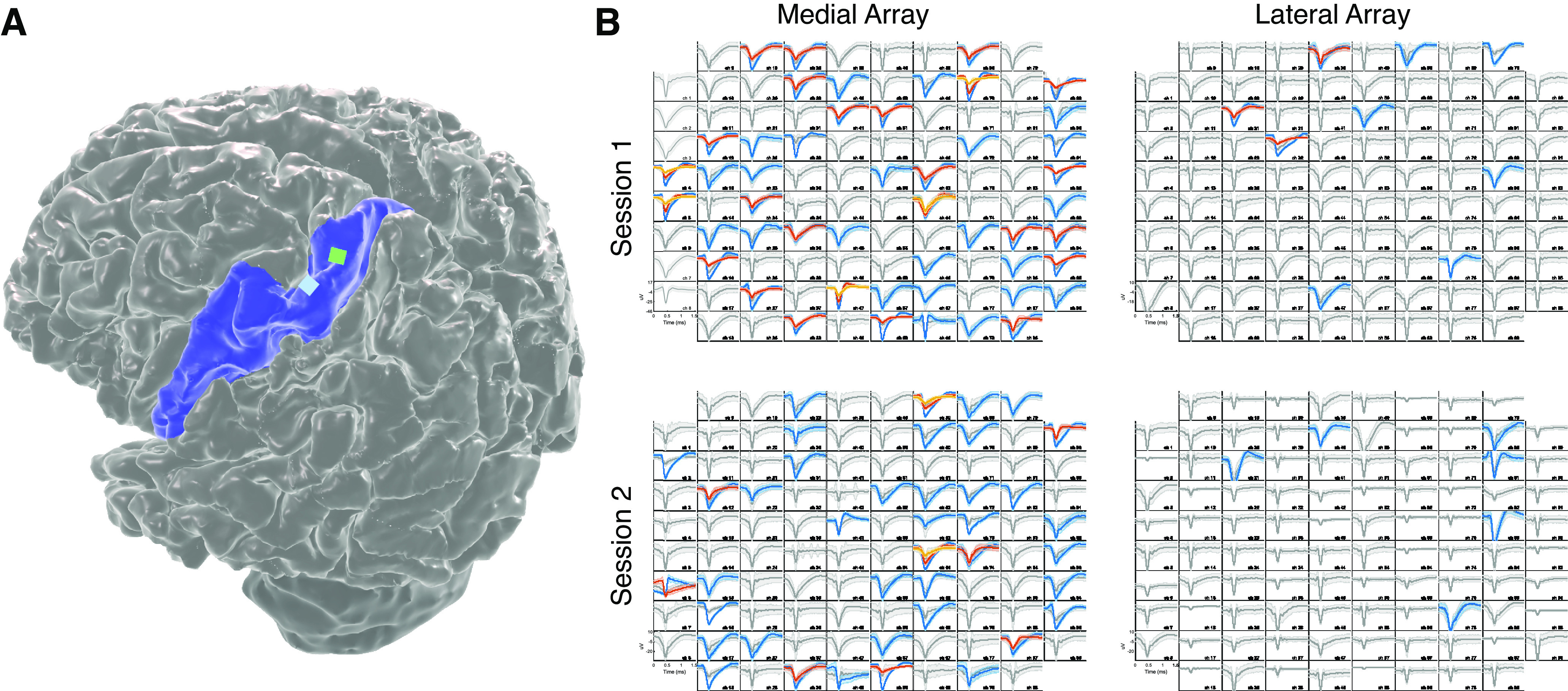
Neural signals for this study were acquired from two microelectrode arrays chronically implanted in left precentral gyrus. ***A***, A three-dimensional reconstruction of the research participants' brain. Blue represents left precentral gyrus. Green and blue squares represent the precise location of the two Utah arrays. ***B***, Spike sorting reveals single-unit and multiunit activity on each array (medial array on the left, lateral array on the right) during the first (top) and second (bottom) recording session. All amplitude plots demonstrate mean ± SD of activity for each threshold crossing event, with color representing event type based on automated sorting algorithm (Vargas-Irwin and Donoghue, 2007). Gray amplitude plots represent multiunit activity. Blue, orange, and yellow amplitude plots represent isolated single-unit activity. There were 78 and 51 single units isolated from the medial array and 12 and 6 single units isolated from the lateral array from Sessions 1 and 2, respectively. The smaller number of single units recorded from the lateral array comports with the finding that activity on the lateral array is typically sparser and less closely associated with task performance.

After a brief system synchronization routine, the participant performed a familiar center-out calibration task to optimize the parameters of the steady-state Kalman filter that would be used for the day's experimental session (Fig. 1*B*) (Malik et al., 2011; Brandman et al., 2018). This calibration task was familiar to the participant, and he was able to complete it quickly and without difficulty; on Session 1, the participant successfully reached and held 63 of 66 presented targets with a mean trial duration of 3.58 ± 1.82 s; and on Session 2, the participant successfully reached and held 95 of 95 presented targets with an average trial duration of 3.09 ± 1.02 s. Of the 384 feature channels (2 arrays with 96 channels per array and two features [spike band power and threshold crossing events] per channel) assessed during calibration, 39 of the 40 feature channels used for decoding for both Session 1 and Session 2 were from the medial array; only 1 feature channel from the lateral array was included in the final version of the steady-state Kalman filter on either session (Fig. 2).

Following a brief rest period, the participant was given the instructions for the memory matching task (Fig. 1*C*). On each trial, the sequential illumination of four brightly colored shapes on the screen indicated the sequence to be matched. The participant was instructed to move the neurally controlled cursor from the center of the screen to each target in the same order as was presented. When he successfully completed the sequence, a bright tone indicated success and the next trial commenced. If the cursor dwelled on an incorrect target or if the participant failed to move the cursor to any target within 5 s, a low-pitched tone indicated trial failure and the next trial commenced. Each session consisted of 10 blocks of 16 trials, with one sequence of four targets per trial. In each block, 12 of 16 trials were of the set “target” sequence for that day, and the other four trials were random distractor sequences. The target sequence was different on the two multiday recording sessions but within a given session was consistent across all blocks.

At each session, the participant quickly engaged in successfully completing the task. At the first session, the participant accurately completed 109 of 120 (90.8%) of the target trials and 33 of 40 (82.5%) of the distractor trials. In the second session, he accurately completed 77 of 120 (64.2%) target trials and 25 of 40 (62.5%) of distractor trials. There was no significant difference in performance between target or distractor trials over the course of 10 blocks on either session (*p* = 0.2504 on Session 1, *p* = 0.84073 on Session 2, Fisher's exact test). On the day following the second session, using the steady-state Kalman filter decoder that had been calibrated for the prior day's session, an abbreviated session of the matching game was performed. The participant accurately completed 33 of 36 target trials (91.7%) and 8 of 12 distractor trials (75.0%).

### Offline replay is observed throughout sleep

**Figure 3.**
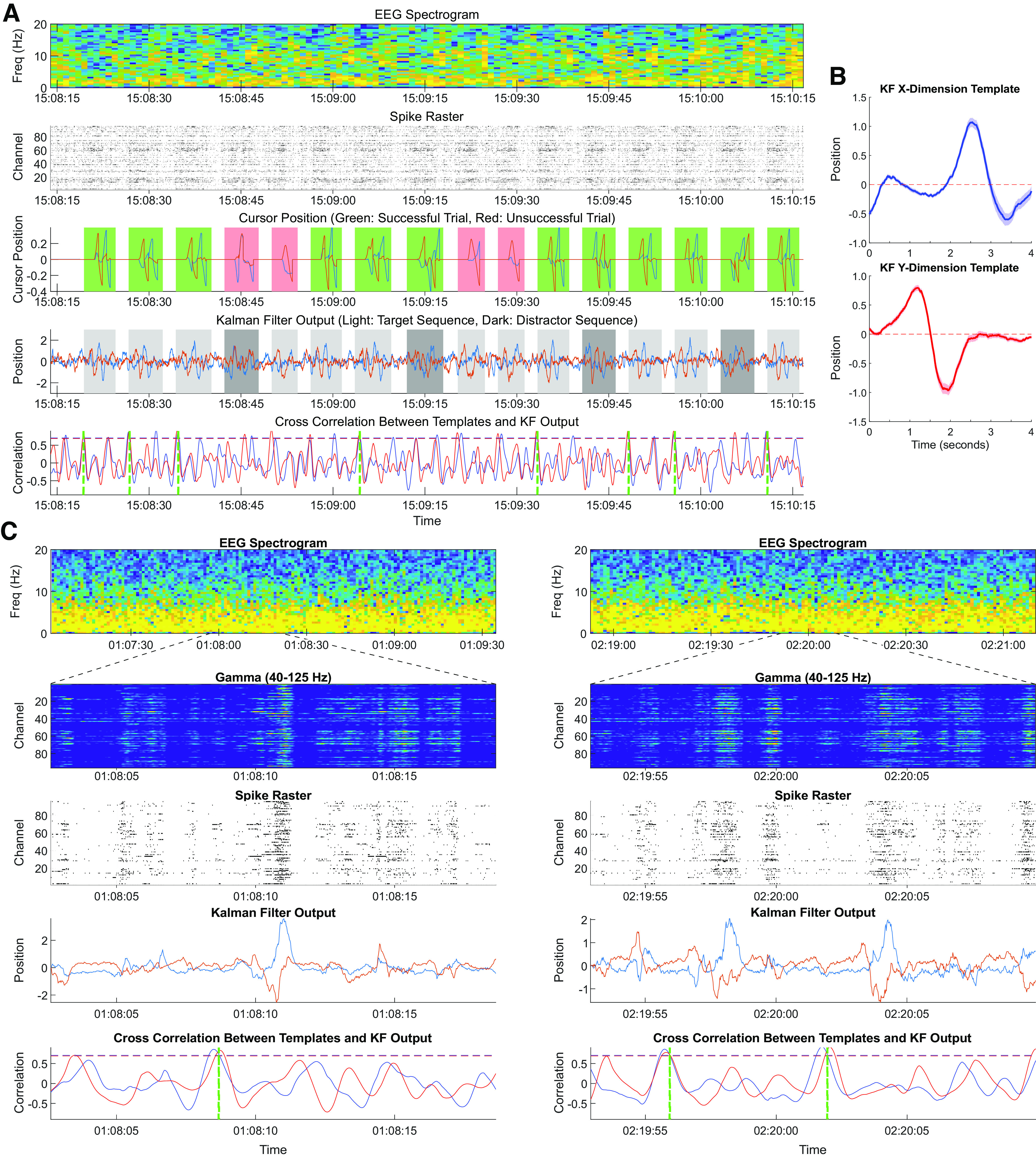
Examples of neural data and processed Kalman filter output during awake task performance and sleep. ***A***, Data from a single experimental block of the motor sequence memory task. Top row, Spectrogram of the surface EEG signal recorded during the block. Second row, Spike raster from the medial PCG multielectrode array, showing increased neural firing during intended movement. Displayed activity is a thresholded, *z*-scored spike raster (with marks indicating times when spiking activity *z* > 1). Third row, Cursor position during each trial of the matching task. Blue line indicates X position. Red line indicates Y position. The cursor is reset to the origin after each successful target acquisition. Green shading represents successful trials. Red shading represents unsuccessful trials. A successful target trial (e.g., Trial 1, at 15:08:20) is the following sequence: up, down, right, left. Target trials indicated by light gray shading on the fourth row; distractor trials are indicated by dark gray shading on the fourth row. Fourth row, The intracortical neural data from the recording session are projected through the Kalman filter to generate a continuous model of intended cursor position (before online filtering/scaling/midpoint correction). Blue line indicates X position. Red line indicates Y position. The intended direction/magnitude of movement is evident even without the online postprocessing applied during active task participation. We used the mean output of the Kalman filter model during successful target trials to generate templates of trial-specific activity. Bottom row, Instantaneous correlation between Kalman filter output and successful target trial template. Blue line indicates X position. Red line indicates Y position. Dashed horizontal lines (blue: 0.71, red: 0.69) indicate the 99th percentile values of the correlation coefficients, which we define as the threshold for a template “match.” Vertical green dashed lines indicate the start time of STCEs, which, though indicated by a discrete event marker on this plot, encapsulate a subsequent trajectory of neural firing. In this example, there are STCEs at 8 of the 9 successful target sequence trials, but at none of the unsuccessful target trials nor during any of the successful or unsuccessful distractor sequence trials. ***B***, Template target trajectories for the Kalman filter output in the X (top) and Y (bottom) dimensions. Templates were generated by aligning and averaging the offline-generated predicted cursor position for each of the successful target trials. The mean values (solid line) are used for subsequent template matching calculations. Shading around the solid line represents the SEM curve. ***C***, Two examples of putative replay events occurring during overnight sleep following the motor task completion demonstrated above from experimental Session 2. Top row, EEG spectrogram showing predominately δ power, indicating a period of SWS. Second row, Relative power in low γ (40-125 Hz) power band from the medial PCG array, demonstrating brief bursts (or ripples) of activity lasting ∼200 ms during sleep. Third row, Spike raster during sleep; as above displaying thresholded, *z*-scored activity from the medial PCG array. Fourth row, Kalman filter output demonstrating replay of 2D trajectory associated with the target task during sleep. Bottom row, Same as in ***A***, the instantaneous correlation between Kalman filter output and successful target trial template. Blue line indicates X position. Red line indicates Y position. Dashed horizontal lines indicate the 99th percentile values of the correlation coefficients which are used to define a threshold “match.” Vertical green dashed lines indicate the time of the STCEs. As above, though indicated by a discrete event marker on this plot, the STCEs encapsulate a subsequent trajectory of neural firing, which is why the stereotyped Kalman filter output trajectory follows the STCE by ∼2 s.

To test the hypothesis that motor cortex replays recently rehearsed patterns of activity during sleep, we used the steady-state Kalman filter used for cursor control during the task blocks as a dimensionality reduction tool to probe for replay in overnight neural activity. For each recording session, we extracted the best-fit parameters for the steady-state Kalman filter from that session's calibration block. Offline, we projected the spiking data from the recording session (both the active task completion blocks as well as the resting/sleeping blocks) through the Kalman filter to produce a time-series of offline predicted cursor position. As a confirmation of this approach, we compared the offline-predicted cursor position to the actual position of the cursor displayed during task performance; as expected, this offline-calculated output of the Kalman filter faithfully reproduced the cursor trajectory (Fig. 3). This served as proof of concept that the offline-recalculated trajectories (which lack the scaling, smoothing, and automatic recentering performed during online task performance) match the trajectories generated during epochs of closed-loop BCI-enabled cursor control. We subsequently used the Kalman filter to generate hypothetical cursor positions during periods of time when there is no cursor being displayed on the screen (i.e., during sleep). Offline Kalman filter activity is generated using the threshold-crossing and spike power data (as described in Materials and Methods) as this is the same neural data used by the closed loop decoding algorithm; we separately examined spike sorted activity on each array to determine the relative contribution of single-unit and multiunit activity to our decoded signals (Fig. 2*B*).

To probe for replay, we aligned and averaged the offline-generated predicted cursor position for both spatial (X and Y) dimensions during each of the successful target trials to create a pair of template target trajectories (Fig. 3*B*). At each time-step of the recording, for each spatial dimension, we calculated the cross-correlation between the target trial template and the actual output of the Kalman filter. This process yielded a pair of time-series of cross-correlations, one for each spatial dimension, equal in length to the duration of the neural recording. We define a “template match” to be a segment of neural recording during which the cross-correlation between template and the Kalman filter output equals or exceeds the threshold correlations calculated. We refer to instances when this threshold is crossed in both the X and Y dimensions as STCEs. As described in Materials and Methods, specific thresholds were derived for each session as the integer percentile that optimized the Youden's J statistic of the STCEs to accurately classify successful target trials. During Session 1, there were 109 successful target trials and 51 other trials. Using correlations above the 98th percentile as threshold yielded 103 STCEs during active task performance, of which 95 correctly identified a successful target trial and 8 occurred during other trials, yielding Youden's J = 0.71; with the correlation threshold at the 99th percentile there were 82 STCEs, of which 79 correctly identified a successful target trial and 3 occurred during other trials, yielding Youden's J = 0.67. During Session 2, there were 77 successful target trials and 83 other trials. Using correlations above the 98th percentile as threshold yielded 104 STCEs during active task performance, of which 65 correctly identified a successful target trial and 39 occurred during other trials, yielding Youden's J = 0.37; with the correlation threshold at the 99th percentile, there were 81 STCEs, of which 61 correctly identified a successful target trial and 20 occurred during other trials, yielding Youden's J = 0.55. Based on these results, for subsequent analyses for Session 1, the 98th percentile was used as the threshold cutoff for template match (cross-correlation threshold values: CC_X_ ≥ 0.7986, CC_Y_ ≥ 0.6870) and for Session 2 the 99th percentile was used (cross-correlation threshold values: CC_X_ ≥ 0.7059, CC_Y_ ≥ 0.6898). By this design, STCEs were observed throughout the game play blocks at the times of most (but not necessarily all) successful target trials but not during distractor trials or unsuccessful target trials (Fig. 3*A*). When occurring during rest or sleep, we refer to these STCEs as putative replay events, as the neural firing, when processed through the Kalman filter, successfully completes the target motor sequence task (Fig. 3; Movies 1, 2, 3).

Movie 1. Two-dimensional Kalman filter output processed from neural data recorded during the afternoon task completion session as the participant was actively engaged in the motor sequence task. Left, Plots show scalp EEG spectrogram, intracortical ripple-band activity, intracortical spike raster, and Kalman filter output. As in Figure 3, blue line indicates X position and red line indicates Y position. Right, Hypothetical 2D cursor position (calculated from offline Kalman filter position) is plotted over time. Reset to the origin after successful target dwell was added manually as these movies are generated from neural data processed offline. Of note, between the three successful target task sequences (starting at approximately 16:27:07, 16:27:15, and 16:27:23), during the actual experimental session, the participant's neural control of the cursor is temporarily paused while he is being shown the cue for the next sequence to match. Thus, the random appearing trajectories traced by neural activity during these brief epochs between successful task completion were not actually displayed on the screen during the actual task completion session. 10.1523/JNEUROSCI.2074-21.2022.video.1

Movie 2. Analogous plots as in Movie 1, but now showing neural activity recorded at 12:50 A.M. as the participant slept. Movie frame rate is slowed down by 50% to facilitate visualization of faster (2-4×) overnight replay events. 10.1523/JNEUROSCI.2074-21.2022.video.2

Movie 3. Analogous plots as in Movie 1, but now showing neural activity recorded at 02:19 A.M. as the participant slept. As above, movie frame rate is slowed down by 50% to facilitate visualization of faster (2-4×) overnight replay events. 10.1523/JNEUROSCI.2074-21.2022.video.3

We then determined when STCEs occurred. In the first session, there were 2 STCEs during the 30 min rest period before beginning the task (4/h), 109 STCEs during the task completion blocks (237/h), 2 STCE during the 30 min rest period immediately after the task completion blocks (4/h), and 66 STCEs during the 9 h of sleep the night after completing the task (7.33/h). In the second session, there were 4 STCEs during the 9 h of sleep the night before completing the task (0.44/h), 0 STCEs during the 30 min rest period before completing the task (0/h), 81 STCEs during the task completion blocks (199/h), 6 STCEs during the 30 min rest period after the task completion blocks (12/h), and 85 STCEs during the 10 h of sleep the night after completing the task (8.5/h).

**Figure 4.**
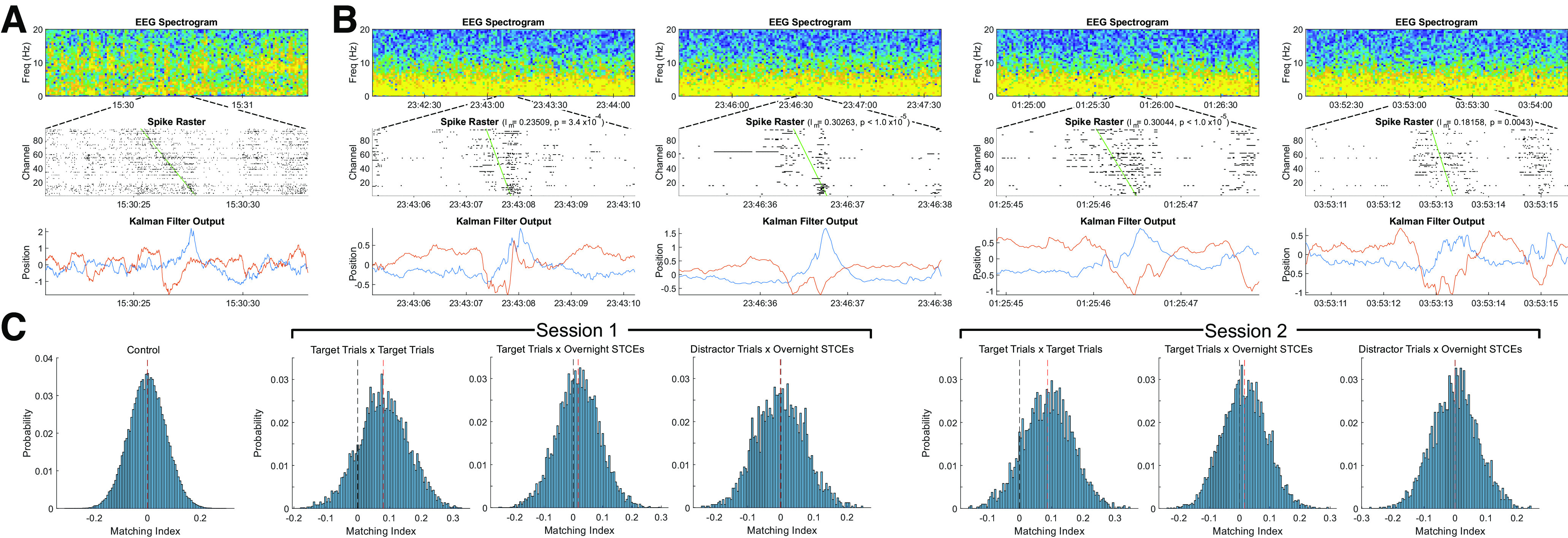
Examples of preserved spiking order during replay events. ***A***, One task completion event from an awake task completion block from the second recording session, serving as a template for the matching indices I_m_ demonstrated in ***B***. Top row, EEG spectrogram showing a normal awake power spectrum with predominant alpha activity. Second row, Thresholded, *z*-scored spike raster (showing as spikes all indices with spiking activity *z* > 1) from the medial PCG array. Channels are ordered from top to bottom in order of timing of peak firing rate during the task completion. Bottom row, Kalman filter output demonstrating 2D trajectory associated with target task completion. ***B***, Four examples of putative replay events occurring during overnight sleep, demonstrating preservation of spiking order during putative replay events. Top row, EEG spectrogram showing predominately δ power, indicating a period of SWS. Second row, *z*-scored spike raster, with channel order as in ***A***, demonstrating preservation of spiking order. Matching index I_m_ and *p* value are indicated in the plot title (compared with control distribution). Green line indicates the best-fit linear regression line between channel order from ***A*** and the timing of the peak firing rate from the replay event being displayed. Bottom row, Kalman filter output demonstrating 2D trajectory associated with the target task completion during sleep. ***C***, Histograms demonstrating the distributions of matching indices during each session; on each plot, the black dashed line indicates 0 and the red dashed line indicates the mean of the distribution. Leftmost panel, Matching indices generated from 100,000 pairs of randomly generated 96 element sequences, which serves as a control distribution. The second histogram represents the distribution of I_m_ across all pairs of successful target trials during Session 1; the mean is significantly greater than the control distribution (*p* < 1 × 10^−100^, Student's *t* test). The third histogram represents the distribution of I_m_ across all pairs of successful target trials and overnight STCEs during Session 1; the mean of this distribution is also significantly greater than the control (*p* = 7.19 × 10^−82^, Student's *t* test). The fourth distribution shows the distribution of I_m_ across all pairs of distractor trials and overnight STCEs during Session 1; the mean of this distribution is not significantly different from the control (*p* = 0.10118, Student's *t* test). The fifth through seventh histograms represent analogous distributions generated during Session 2; the distributions of I_m_ across all pairs of successful target trials and all pairs of successful target trials with overnight STCEs are both significantly greater than the control distribution (target trials vs target trials: *p* < 1 × 10^−100^, Student's *t* test; target trials vs overnight STCEs: *p* = 1.17 × 10^−77^, Student's *t* test). The distribution of I_m_ across all pairs of distractor trials and overnight STCEs is again not significantly different from the control (*p* = 0.96093, Student's *t* test).

By calculating the matching indices I_m_, we found that the spiking sequence was preserved between target task completion and overnight STCEs significantly greater than expected by chance (Fig. 4). During Session 1, the distribution of I_m_ across all pairs of successful target trials (*n* = 109, mean ± 95% CI I_m_: 0.080 ± 0.0020) was significantly greater than a control distribution (mean ± 95% CI I_m_: 0.00015 ± 0.00043, *p* < 1 × 10^−100^, Student's *t* test). Similarly, the distribution of I_m_ across all pairs of successful target trials and overnight replay STCEs (*n* = 66, mean ± 95% CI I_m_: 0.016 ± 0.0017) was also significantly greater than the control distribution (*p* = 7.19 × 10^−82^, Student's *t* test). However, the distribution of I_m_ across all pairs of distractor trials (*n* = 40) and overnight replay STCEs was not significantly greater than the control distribution (mean ± 95% CI I_m_: −0.0021 ± 0.0029, *p* = 0.10118, Student's *t* test). As expected, the mean of the control distribution (described in Materials and Methods) is not significantly different from 0 (*p* = 0.4889, Student's *t* test). For Session 2, the distribution of I_m_ across all pairs of successful target trials (*n* = 77, mean ± 95% CI I_m_: 0.088 ± 0.0028) was significantly greater than the control distribution (*p* < 1 × 10^−100^, Student's *t* test). Similarly, the distribution of I_m_ across all pairs of successful target trials and overnight replay STCEs (*n* = 85, mean ± 95% CI I_m_: 0.017 ± 0.0018) is significantly greater than a control distribution (*p* = 1.17 × 10^−77^, Student's *t* test). As in Session 1, the distribution of I_m_ across all pairs of distractor trials and overnight replay STCEs was not significantly greater than the control distribution (mean ± 95% CI I_m_: 0.00021 ± 0.0024, *p* = 0.96093, Student's *t* test).

**Figure 5.**
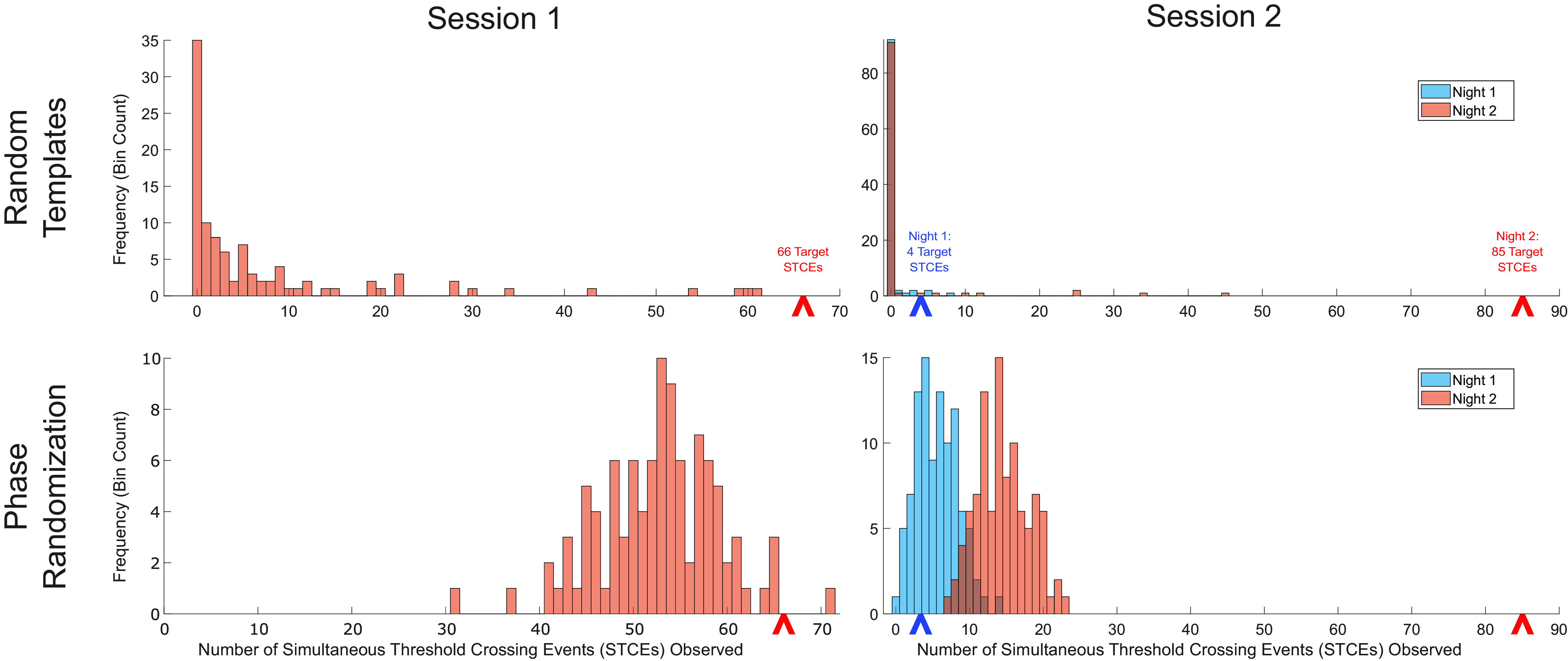
Controls demonstrating expected number of STCEs by chance versus actual number of putative replay events observed. Top left, Histogram represents the number of Session 1 overnight STCEs observed when matched on 100 pseudo-templates generated from randomly selected epochs of neural data recorded during the afternoon pretask rest session. These data are meant to represent the distribution of replay events that would be expected by chance. The number of putative replay events of the target templates observed overnight (66) is greater than would be expected by chance (*p* = 0.019802, Mann–Whitney *U* test). Top right, Histogram represents the number of Session 2 STCEs observed (again matched on 100 pseudo-templates generated from randomly selected epochs of neural data recorded during the afternoon pretask rest session) during the night before (blue bars) and after (orange bars) performing the motor sequence task. The number of STCEs observed the night before task performance (4) is not statistically different from would be expected by chance (*p* = 0.079208, Mann–Whitney *U* test); the number of STCEs observed the night after task performance (85) is significantly different from would be expected by chance (*p* = 0.019802, Mann–Whitney *U* test). The number of STCEs observed by chance is not different between the two nights (*p* = 0.61606, Mann–Whitney *U* test). Bottom left, Histogram represents the number of Session 1 overnight STCEs observed using the target template matched against Kalman filter output that has been randomized in phase in 5 min segments. This phase-randomized Kalman filter output effectively preserves all underlying neural spike statistics while shuffling the relative sequences of firing. The number of overnight putative replay events is significantly greater than the number of STCEs observed in the phase-randomized data set (*p* = 0.041577, *t* test). Bottom right, Histogram represents the number of Session 2 STCEs observed using the target template matched against the phase-randomized Kalman filter output during the night before (blue bars) and after (orange bars) performing the motor sequence task. The number of STCEs observed the night before task performance is not significantly different from the number expected by chance (*p* = 0.57529, *t* test); the number of STCEs observed the night after task performance is significantly greater than the number expected by chance (*p* = 3.2698 × 10^−36^_,_ *t* test). The number of STCEs observed by chance the night after task performance is significantly greater than the number of STCEs observed by chance the night before task performance (*p* = 9.7723 × 10^−49^_,_ *t* test).

To determine whether the number of STCEs observed the nights after task completion was greater than expected by chance, we performed a series of controls. First, for each session, we generated a distribution of expected number of STCEs for nonsalient neural patterns by randomly selecting 100 segments of neural activity, each equal in duration to the target sequence templates, from the 30 min rest period before the task blocks. We processed each of these segments of data through the Kalman filter to generate 100 pairs of pseudo-templates. For each pseudo-template pair, we repeated the template-matching procedure described above, calculating the cross-correlation between the template and the Kalman filter output at each time-step of the recording, and counted the number of STCEs observed overnight for each pseudo-template (Fig. 5). For Session 1, the number of target template STCEs observed overnight (66) was significantly greater than the number expected by chance (median (interquartile range [IQR]) = 2 (0-8.5), *p* = 0.019802, Mann–Whitney *U* test). For Session 2, the number of target template STCEs observed during the night after task completion (85) was also significantly greater than the number expected by chance (median (IQR) = 0 (0-0), *p* = 0.019802, Mann–Whitney *U* test). For Session 2, the number of target template STCEs observed during the night before task completion (4) was not significantly greater than expected by chance (median (IQR) = 0 (0-0), *p* = 0.079208, Mann–Whitney *U* test). For the second multiday recording session, there was no difference in the number of STCEs expected by chance between the night before and after task completion (*p* = 0.61606, Mann–Whitney *U* test). Of note, based on the number of bootstrap iterations performed, 0.019802 is the smallest *p* value obtainable by the Mann–Whitney *U* test.

In a second bootstrapping procedure, we assessed for the selectivity of neural firing sequence while controlling for the underlying population firing rate statistics. Separately for each multiday session, for each of 100 bootstrap iterations, the Kalman filter output of the neural recording was segmented into 5 min epochs. Within each epoch, we randomized the phase of the X and Y dimension of the Kalman filter output. We then reassembled the epochs into full-time-series and performed the cross-correlation matching procedure described above using the successful trial target templates (Fig. 5). For Session 1, the number of target template STCEs observed overnight (66) was significantly greater than the number observed in the unstructured phase-randomized control distribution (median (IQR) = 53 (48-57), *p* = 0.041577, Student's *t* test). For Session 2, the number of target template STCEs observed the night after task completion (85) was also significantly greater than the number in the unstructured phase-randomized control distribution (median (IQR) = 14 (12-17), *p* = 3.2698 × 10^−36^_,_ *t* test). For Session 2, the number of target template STCEs observed during night before task completion (4) was not significantly greater than number observed in the control distribution (median (IQR) = 5.5 (3-8), *p* = 0.57529). Within the phase-randomized control subspaces, there were significantly more STCEs the night after than the night before task completion (median (IQR) = 5.5 (3-8) the night before; 14 (12-17) the night after; *p* = 9.7723 × 10^−49^_,_ *t* test).

As it also served as a control for chance occurrences of replay events, we again note that, for the second recording session, we were able to record neural activity during both the night before and after the task completion session. As mentioned above, there were four STCEs during the 9 h of sleep the night before completing the task (0.44/h) and 85 STCEs during the 10 h of sleep the night after completing the task (8.5/h).

### Replay occurs with temporal compression

**Figure 6.**
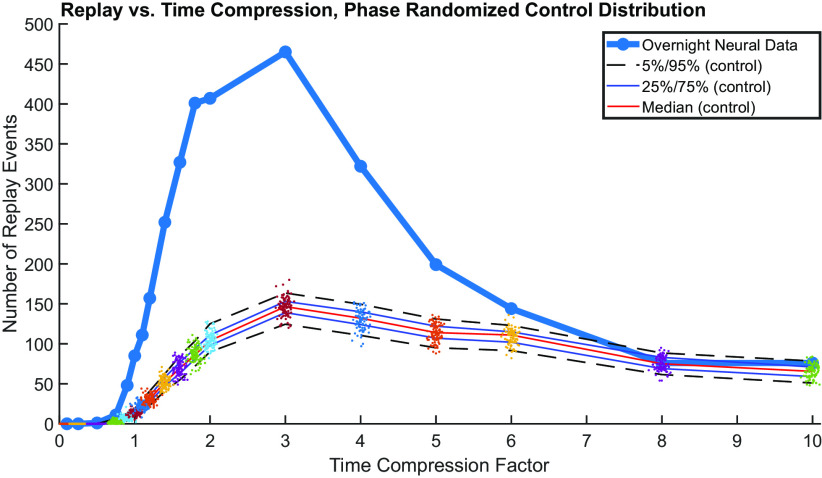
Number of putative replay events observed overnight versus the speed of replay. Bold dark blue represents the number of replay events at each replay speed (Night 2, recording Session 2). At each replay speed, a control distribution of number of STCEs was generated using the phase-randomized control bootstrapping procedure described above. All data points are demonstrated, with median, 25%/75%, and 5%/95% limits of the control distribution indicated on each plot. Although the number of STCEs observed by chance also increased with increasing τ (because shorter patterns of activity are more likely to occur by chance), the number of replay events was nonetheless significantly greater than expected by chance over a wide range of replay speeds.

We next assessed whether there was evidence of replay events occurring at increased or decreased speeds compared with the speed of the original task (Euston et al., 2007). For each recording session, we fit the target sequence Kalman filter templates using cubic splines. We then adjusted the duration of the time axis of the templates by a factor τ to alter the duration of the templated replay event, such that, for example τ = 2, the four-target sequence is replayed in half as much time. We varied τ over 18 values from 0.1 to 10. For each pair of templates at each value of τ, we repeated the correlation matching paradigm described above, noting the time and number of STCEs. Because templates of shorter duration were more likely to appear in the neural recording by chance, we repeated the phase-randomized bootstrap control described above for each value of τ to establish a reference distribution and assess for statistical significance. In Session 2, we found that the number of putative replay events observed increased substantially from 1× to 6× (Fig. 6). The number of STCEs observed by chance also increased with decreasing template duration (i.e., increasing τ), but the number of replay events after Session 2 was nonetheless significantly greater than expected by chance over a wide range of replay speeds. The number of replay events observed at faster speeds after Session 1 was also increased but did not reach statistical significance.

### Replay is influenced by sleep stage

**Figure 7.**
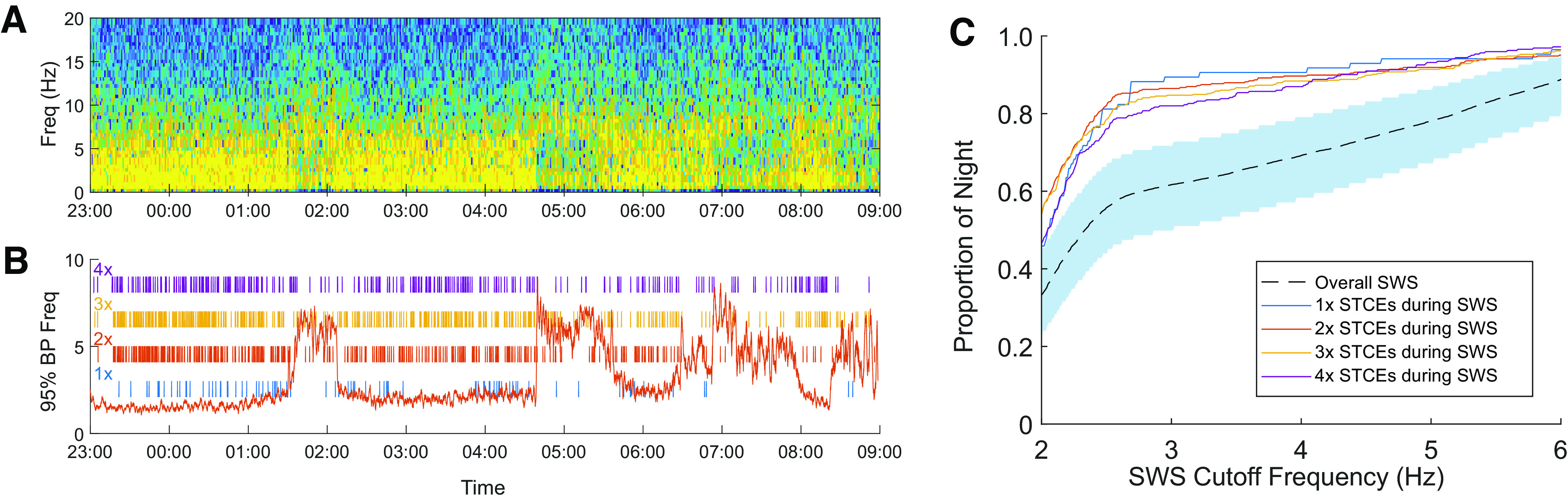
Timing of replay events over the course of the night: Session 2, Night 2. ***A***, Scalp EEG spectrogram overnight demonstrates epochs of predominately δ activity, initially 90-120 min in duration but shortening over the course of the evening, with intervening epochs lasting 15-30 min of higher-frequency predominant activity, consistent with a typical night of human sleep. ***B***, Red line indicates the 95% FPL of the EEG spectrogram shown in ***A***; the value of this metric used as a threshold for SWS is varied systematically over a range of 2-6 Hz. Individual putative replay events are demonstrated as raster marks on the same time axis for replay at 1× (blue), 2× (red), 3× (yellow), and 4× (purple). ***C***, The proportion of the night spent in SWS (dashed line) or proportion of putative replay events that occurred during SWS as a function of SWS-defining cutoff threshold. As the threshold defining SWS increases, both the proportion of the night defined as SWS as well as the number of replay events categorized as occurring during SWS tautologically increase. Over the range of threshold FPLs used to define SWS, the number of putative replay events that occur during SWS is significantly greater than the number of events that would be expected if replay events occurred randomly throughout the night (and were thus simply proportional to the fraction of the night spent in SWS). Shaded blue region represents a 95% CI (based on a binomial distribution) for the proportion of putative 1x replay events expected to be observed during SWS if simply proportional to the fraction of the night spent in SWS (95% CIs for 2×, 3×, and 4× replay events all fall within this shaded region as there are more overall replay events at these faster speeds).

To assess for a relationship between cortical replay and sleep stage, we quantified the dominant frequency in the scalp EEG recording by calculating at each time-step of the 95% FPL, which we define as the frequency below which contains 95% of the power in the EEG spectrogram. The overnight recording session was then segmented into periods of SWS versus non-SWS based on a threshold FPL. This threshold was varied systematically from 2 Hz (a strict definition of SWS) to 6 Hz (a strict definition of non-SWS). To determine whether STCEs occurred more frequently during SWS than would be expected by chance, at each FPL threshold, we calculated the proportion of the overnight recording that was classified as SWS versus non-SWS, as well as the number of STCEs that occurred during SWS versus non-SWS. Using a two-sample Kolmogorov–Smirnov test with the null hypothesis that the number of STCEs observed during SWS is simply proportional to the fraction of the night spent in SWS, we found that, over the range of FPL thresholds investigated, the distribution of the number of putative replay events observed during SWS was significantly greater than the number anticipated (for Session 1: *p* = 5.81 × 10^−10^ for 1× replay events; for Session 2: *p* = 2.48 × 10^−118^ for 1× replay events, *p* = 5.32 × 10^−103^ for 2× replay events, *p* = 1.21 × 10^−85^ for 3× replay events, *p* = 6.80 × 10^−70^ for 4× replay events, two-sample Kolmogorov–Smirnov test) (Fig. 7). For Session 1, replay events at faster speeds (2×, 3×, 4×) were not significantly more likely to occur during SWS than non-SWS.

### Replay occurs during cortical ripples

**Figure 8.**
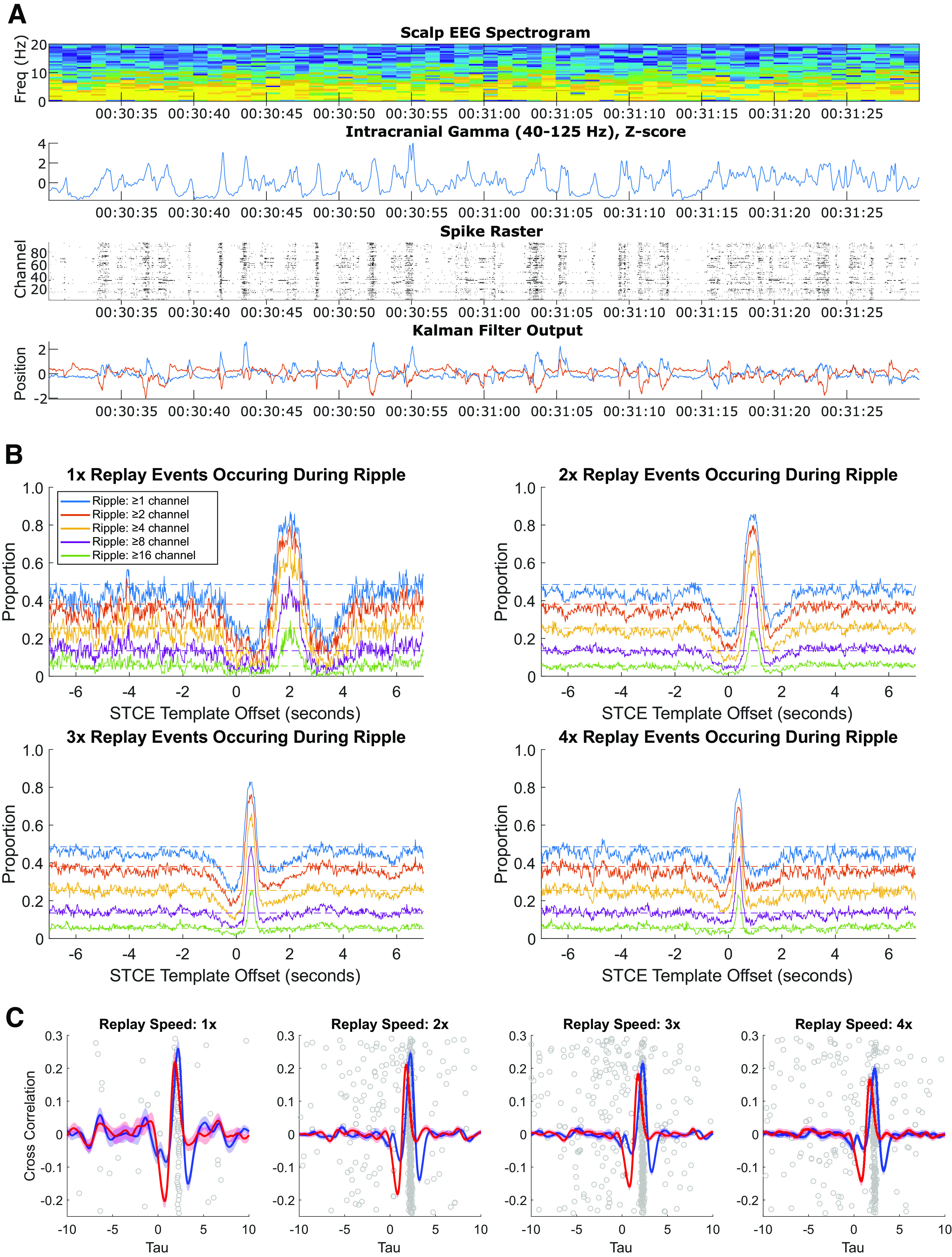
Relationship between replay events and ripple activity. ***A***, Example of neural activity during an epoch of sleep. Top row, Scalp EEG spectrogram represents power predominately in the δ band. Second row, Average power in the ripple band (40-125 Hz) of the LFP from the medial intracortical array (*z*-scored within a 1 min sliding window) to identify peaks in the ripple-band activity. Third row, Intracortical spike raster from the medial array across all 96 channels. Fourth row, Kalman filter output represents frequent deflections in hypothetical cursor position most prominently during bursts of activity aligned with peaks in the ripple-band LFP. ***B***, Replay events (STCEs) are aligned in time with ripples. Five different ripple magnitudes were examined, each quantified by the number of channels on which a ripple was simultaneously observed (of the 96 channels on the medial array). For each threshold, for each of four replay event speeds (1×, 2×, 3×, 4×), we examined what proportion of the overall number of STCEs (recorded on Night 2 of Session 2) occurred during a period classified as a ripple. Because each STCE is point process representing a trajectory of activity, the peak in neural activity lags the STCE by fixed period. To account for this lag, we varied the timestamp of all STCEs in 20 ms time-steps from −7 to 7 s and recalculated the proportion occurring during ripple on each iteration. On each plot, dashed horizontal lines indicate the baseline proportion of overnight sleep classified as ripple based on each threshold [0.49 for ripple on ≥ 1 channel (blue); 0.38 for ripple on ≥ 2 channels (red); 0.25 for ripple on ≥ 4 channels (yellow); 0.13 for ripple on ≥ 8 channels (purple); 0.05 for ripple on ≥ 16 channels (green)]. For each replay speed, regardless of ripple channel threshold, there is a clear peak in the proportion of replay events that occur during an epoch defined as a ripple that occurs at an offset equal to approximately half the template duration. As expected, the peak offset decreases monotonically with increasing replay speed. In a window around each peak, there is a refractory period during which a replay event is less likely than baseline to be observed. ***C***, Cross-correlation between the Kalman filter template matching signal and the number of channels observed to have ripple activity in a ±5 s window around each putative replay event. Mean cross-correlation for the X dimension (red) and Y dimension (blue) ±95% CI is shown for each replay speed. On each plot, for each putative replay event, the time of the peak in the sum of the cross-correlograms for both the X and Y dimensions is shown as gray circles along the *x* axis; position along the *y* axis is randomly scattered to allow for visualization of each data point. The median time to the peak is 2.2 s for each replay speed examined.

Prior work has demonstrated that replay frequently occurs during brief bursts of coordinated activity referred to as SWRs (Buzsáki, 2015). In several previous studies, LFP power within the low γ range (∼40-125 Hz, with a peak in human cortex at 80 Hz) has been shown to robustly capture ripple events in hippocampus and cortex (Staresina et al., 2015; Jiang et al., 2017, 2019; Dickey et al., 2021a). To probe for a relationship between replay and ripples within our data, we first quantified the power from the intracortical arrays within this broadly defined “ripple-band” and compared peaks in activity to the timing of replay events (Fig. 8*A*). Having observed a clear qualitative relationship between peaks in the ripple-band and replay events, we more rigorously probed the intracortical data for ripples using the automated technique described by Jiang et al (Jiang et al., 2019; Dickey et al., 2021a, 2021b). As described in Materials and Methods, individual segments of data, bandpass filtered at 60-100 Hz and at least 40 ms in duration, that were in the top 10th percentile for RMS magnitude and confirmed to have at least three distinct peaks are characterized as ripples. Ripples were characterized on each channel independently, allowing us to additionally quantify the magnitude of a ripple event by the number of simultaneous channels on which rippling activity is observed; we considered five threshold values (1, 2, 4, 8, or 16 channels with simultaneous ripple activity) of ripple magnitude.

For each threshold value and for each of four replay event speeds (1×, 2×, 3×, 4×), we then calculated the proportion of the overall number of overnight STCEs that occurred during a ripple. As each STCE is point process (corresponding to the time of a simultaneous peak in the cross correlations between each dimension of the Kalman filter output and the idealized template) that represents a trajectory of activity that evolves over time, the peak in neural activity (and subsequently Kalman filter position magnitude) is expected to lag the STCE by a fixed period of time (approximately half the STCE template length). To account for this feature of the data, we systematically varied the timestamp of each STCE in 20 ms steps from −7 to 7 s around the original STCE and iterated the above analysis. We then compared the proportion of STCEs that occurred during a ripple at each offset to the background rate at which ripples occurred. If replay events were unrelated to ripples, we would expect the proportion of STCEs that occur during ripples to be comparable to the background rate of ripples, regardless of any lag introduced between the time of onset of the replay event and the time of the ripple. In contrast, we find that there is a clear peak in the proportion of replay events that occur during an epoch defined as a ripple at a time offset approximately half the duration of STCE template (Fig. 8*B*). As expected, the peak offset decreases monotonically with increasing replay speed, as template length is shortened by the same factor. In a temporal window around each peak, there is a refractory period during which a replay event is less likely than baseline to be observed.

As a second test of the temporal concordance between putative replay events and cortical ripples, separately for the X and Y Kalman filter dimensions, we calculated the cross-correlation between the Kalman filter template matching signal (itself the cross-correlation between the target template and the continuous steady-state Kalman filter output) and the number of channels observed to have ripple activity in a ±5 s window around each putative replay event (Fig. 8*C*). Across all putative replay events, the median time to the peak in the sum of these curves was 2.2 s, suggesting that the number of channels with ripple activity peaks ∼2.2 s after the start of a replay event. In this analysis, the time to peak of these cross-correlograms does not decrease at faster speeds, as the initial cross-correlation (between the target template and continuous Kalman filter output) normalizes the period between STCE onset and the peak in activity for faster replay events.

## Discussion

We find that the sequence of neural activity in human motor cortex recently used to complete a novel motor task via an intracortical BCI (iBCI) is replayed during sleep. We performed dimensionality reduction by using the Kalman filter used for signal decoding during iBCI-enabled cursor movement to demonstrate cortical replay of a recently rehearsed motor sequence in the absence of any observable external input or feedback. This is the first work, to our knowledge, to directly demonstrate the replay of task-specific neural activity sequences in human motor cortex during sleep. Prior work has demonstrated that replay likely plays an important role in memory consolidation in rodent hippocampus and neocortex, (Skaggs and McNaughton, 1996; Euston et al., 2007; Ji and Wilson, 2007; Singer et al., 2013; Gulati et al., 2014; Buzsáki, 2015; Ramanathan et al., 2015), and more recent work demonstrates evidence of replay in human neural systems (Eichenlaub et al., 2020; Vaz et al., 2020). These findings, taken together with the results reported herein, suggest that replay may be a conserved mechanism of learning and memory across multiple cortical systems, including semantic, navigational, and motor systems, and further supports the role of sleep in memory consolidation (Huber et al., 2004; Peigneux et al., 2004; Grosmark et al., 2012; Jiang et al., 2017; Zhang et al., 2018).

This work expands on our previous findings demonstrating indirect evidence of motor cortical replay during a resting state after task performance (Eichenlaub et al., 2020). Because of technical limitations, that prior study was unable to examine intracortical neural activity during overnight sleep. With advances in our wireless recording system, we can now obtain intracortical signals throughout the night (Simeral et al., 2021). We find that putative replay events occur throughout the night, are more common during sleep with increased slow-wave activity, and occur during epochs consistent with ripple activity.

Consistent with prior studies, after one of the sessions, we found that target sequences were frequently replayed faster than observed during the awake behavior (Euston et al., 2007; Davidson et al., 2009). The behavioral significance of temporal compression is uncertain; one well-supported hypothesis suggests it reflects the ratio between the behavioral timescale and some intrinsic network time constant. Rodent studies have shown that the temporal compression ratio of hippocampal replay varies inversely with behavioral task duration, such that neural sequences associated with navigation on a small track are typically replayed at 4-6×, whereas those associated with task performance on a larger track may be replayed at up to 15-20× (Lee and Wilson, 2002; Euston et al., 2007; Davidson et al., 2009). In our study, we found replay occurring at multiple time scales, and although more common at faster speeds, our statistical approach does not allow us to state whether a particular temporal compression ratio was dominant. Significantly, our dimensionality reduction tool in this investigation, the Kalman filter, has its own intrinsic time constant (Malik et al., 2011). Thus, we cannot be certain if the decline in replay at higher speeds (e.g., 4-10×) is actually because of the absence of faster replay or simply an inability to translate faster neural sequences into a kinematic representation. Future work using target sequences of varying duration will test this hypothesis directly. Furthermore, we note that statistically significant temporal compression was only observed overnight after one of the task performance sessions; replay in general was more robust after this second session, and as we describe below, this may have been because of increased perceived task difficulty on this second session; future work in which task difficulty is systemically varied will help to clarify this point as well.

Interestingly, recently published human intracortical recordings demonstrate replay without evidence of temporal compression (Vaz et al., 2020). In that study, replay was observed in awake participants that were instructed to learn the association between word pairs. As these participants were performing a primarily verbal, rather than motor, memory task, this raises the possibilities that temporal compression of replay is either specific to sleep, motor learning, or, as mentioned above, a consequence of temporal constraints imposed by the physical task. Replay of motor tasks has also been observed in the awake state in animal models, but these studies did not specifically address the question of replay speed (Karlsson and Frank, 2009; Carr et al., 2011; Singer et al., 2013). Additionally, in this verbal recall task, replay was observed as participants were actually performing a task (i.e., stating the recalled word); thus, temporal compression may have been suppressed for the purpose of generating the appropriate speech output. In contrast, in our study, constraints on cursor speed placed by the time constant of the Kalman filter are removed in the sleeping state, and so the observation of fastforward replay is consistent with literature demonstrating the ability of cortical processing to increase replay speed in the absence of physical limitations on task performance.

If temporal compression results from the ratio between the behavioral timescale and an intrinsic neuronal network time constant, a promising candidate is the SWR. In rat hippocampus, both awake and sleep replay is observed predominately within the spiking activity of SWRs (Wilson and McNaughton, 1994; Carr et al., 2011, 2012; Singer et al., 2013; Papale et al., 2016); and in humans, hippocampal SWRs are correlated with population activity changes in cortex that support memory consolidation during SWS (Jiang et al., 2019). Electrical or optogenetic disruption of SWRs impairs spatial memory consolidation (Girardeau et al., 2009), disrupts memory in novel more so than familiar tasks (van de Ven et al., 2016), and is rewarded more so than unrewarded conditions (Michon et al., 2019), suggesting a role for replay most significantly in conditions of novel or rewarded tasks, features linked with behavioral/memory saliency. Given the finite duration of SWRs, it is interesting to consider whether these events provide the upper limits on a “quanta” of memory, serving as the electrophysiologic correlate of the well-established “7 ± 2” limit observed in psychophysics (Kamiński et al., 2011). It will again be interesting to observe the interplay of replay duration, temporal compression, and task performance in studies with prolonged task duration.

We see a preponderance of replay events occurring during SWS, which has been strongly implicated in memory consolidation and learning. Human studies have demonstrated that a local increase in slow-wave activity is associated with improvement in task performance following sleep (Huber et al., 2004). As mentioned previously, SWRs are predominant during slow-wave, non-REM sleep (Miyawaki and Diba, 2016). Human intracranial EEG recordings also provide evidence of replay tied to hippocampal SWR during nREM sleep (Zhang et al., 2018). In contrast, there is evidence that REM sleep serves as a preparatory “unlearning” stage, during which synaptic energy stores are replenished and synaptic weights modified to promote subsequent learning during awake and nREM states (Grosmark et al., 2012), although exploring this is outside the scope of our current work.

Surprisingly, our participant was more accurate during the first recording session than the second. The reason for his decline in perceived proficiency may be somewhat counterintuitive but was readily apparent during the experimental recording sessions. During the first recording session, the difficulty of the iBCI-simulated motor task was appropriately matched to his proficiency with two-dimensional cursor control. Recording session two, however, took place 9 months later, during which time the participant acquired additional facility and dexterity in two-dimensional cursor control. Consequently, during the second multiday recording session, he often moved the cursor too quickly and failed to dwell on the targets long enough to register before moving to the next one, resulting in several unsuccessful trials. He needed to be reminded more than once to “slow down” during the second session. With practice his performance did improve, and he performed better the following morning when the task was repeated. If replay is believed to be a marker or mechanism supporting skill acquisition, the greater difficulty encountered during task performance on the second session may explain the increased amount of replay after the second session compared with the first session; this may also account for the statistically significant replay at faster speeds observed following the second session. We also unexpectedly found that within the phase-randomized control populations, there were more STCEs observed by chance the night after task performance than the night before (Fig. 4, bottom right). We suspect that the day of task performance may have constrained or otherwise altered the high-dimensional space encapsulating the overnight neural activity, resulting in a greater number of STCEs occurring by chance through the pseudo-randomization procedure used (though still statistically far fewer than were actually observed experimentally).

The most significant limitation of this work is that it was performed in a single participant. Because of the nature the recording preparation (as well as restrictions placed on human subjects research that were present for large portions of 2020), we have not yet been able to perform these studies, which involve continuous overnight recording from intracortical microelectrode arrays chronically implanted in human motor cortex, in multiple participants. We are hopeful that future studies will be able to reproduce and expand on these findings. Nonetheless, we believe that the validity of these findings is supported by their statistical robustness as well as the consistency of our findings with prior literature, including the presence of temporal compressed replay, evidence of interaction between replay and ripples, and preponderance of replay events during SWS. A second limitation regards the single-channel EEG, which limits our ability to catalog certain electrophysiologic features of sleep and formally stage the sleep recorded. Future work will use a more robust EEG preparation to allow for both precise sleep staging and the opportunity to explore the relationship between scalp EEG electrographic phenomena, such as sleep spindles and K-complexes, and intracortical activity.

In conclusion, using an intracortical brain computer interface paired with wireless EEG, we demonstrate evidence of offline replay of neural activity in human motor cortex during SWS. These findings support models of learning and memory based on offline replay and provide evidence of engagement of motor cortical circuitry in memory processing during SWS.
